# Computational Identification and Analysis of the Key Biosorbent Characteristics for the Biosorption Process of Reactive Black 5 onto Fungal Biomass

**DOI:** 10.1371/journal.pone.0033551

**Published:** 2012-03-19

**Authors:** Yu-Yi Yang, Ze-Li Li, Guan Wang, Xiao-Ping Zhao, David E. Crowley, Yu-Hua Zhao

**Affiliations:** 1 College of Life Sciences, Zhejiang University, Hangzhou, People's Republic of China; 2 Taizhou Municipal Hospital, Taizhou, People's Republic of China; 3 Department of Environmental Science, University of California Riverside, Riverside, California, United States of America; University of Houston, United States of America

## Abstract

The performances of nine biosorbents derived from dead fungal biomass were investigated for their ability to remove Reactive Black 5 from aqueous solution. The biosorption data for removal of Reactive Black 5 were readily modeled using the Langmuir adsorption isotherm. Kinetic analysis based on both pseudo-second-order and Weber-Morris models indicated intraparticle diffusion was the rate limiting step for biosorption of Reactive Black 5 on to the biosorbents. Sorption capacities of the biosorbents were not correlated with the initial biosorption rates. Sensitivity analysis of the factors affecting biosorption examined by an artificial neural network model showed that pH was the most important parameter, explaining 22%, followed by nitrogen content of biosorbents (16%), initial dye concentration (15%) and carbon content of biosorbents (10%). The biosorption capacities were not proportional to surface areas of the sorbents, but were instead influenced by their chemical element composition. The main functional groups contributing to dye sorption were amine, carboxylic, and alcohol moieties. The data further suggest that differences in carbon and nitrogen contents of biosorbents may be used as a selection index for identifying effective biosorbents from dead fungal biomass.

## Introduction

Large amounts of dyes are extensively used in the textile, leather, cosmetics, plastic, food, and pharmaceutical industries, in which many dyes are classified as both toxic to human health and harmful to aquatic ecosystems [1]. Therefore, many different treatment methods have been developed and used for dye removal, including chemical precipitation, reverse osmosis, ozonation, membrane filtration and photodegradation [2]. As many dye product industries are located in developing countries, high capital and operational costs or secondary sludge disposal problem has hindered the practical application of many of these methods [3]. Adsorption has been found to be one of the most efficient and low cost techniques for treating dye effluents. Adsorption can handle large flow rates, producing a high-quality effluent that does not result in the formation of harmful substances [4], [5]. Among the different materials studied to date, activated carbon is one of the most effective adsorbent for dyes. However, this adsorbent is relatively expensive and presents problems with regard to final disposal [6]. Alternative adsorbents have been proposed and are now being widely investigated. These include natural materials derived from waste materials from industry and agriculture, as well as biosorbents that are produced from microbial biomass. The latter are often found to be even more selective than traditional ion-exchange resins and activated carbons, and potentially provide an inexpensive method for dye removal [7].

Fungal biomass is a relatively inexpensive biosorbent that can be produced using simple fermentation techniques and inexpensive growth media. Prior studies with this material has shown that fungal biomass can be used been for removal of heavy metals, dyes and polycyclic aromatic hydrocarbons [8], [9], [10]. Dead fungal biomass could be further divided into two main forms in more specific: powdered fungal biomass [11] and fragmental fungal biomass [12]. Powdered fungal biomass loses almost all characteristics of fungal cell structures, whereas fungal cell structures could still be identified in fragmental fungal biomass. The mechanisms of biosorption involved surface adsorption/precipitation and intracellular accumulation depending on the location of the adsorbate [13]. With powdered fungal biomass, surface adsorption is likely mediated by ionic, chemical, and physical interactions.

Reactive Black 5 was selected in this study as a model anionic azo dye for adsorption experiment with its stability under different pH [14], [15], [16], which was abundantly used in textile industries for dyeing and found to be moderately toxic to *Vibrio fischeri* [17]. Nine strains of fungi were selected in this study, which belonged to ascomycota, basidiomycota and zygomycota, respectively and widely used as biosorbents for pollutants removal. In this research, the main aim was to investigate biosorption mechanisms for Reactive Black 5 on to powdered fungal biomass and the role of textural and chemical element compositions on adsorption capacities. Firstly, adsorption kinetics and isotherms were used to model the experimental data to discover the biosorption mechanisms. Then, a constructed artificial neural network was used to model the influence of differences in their textural and chemical element compositions on adsorption capacities and to identify the key physicochemical parameters that affected the biosorption process.

## Materials and Methods

### Materials and Microorganisms

Reactive Black 5 was purchased from Sigma-Aldrich Company and its detail information was shown in Table S1. Potato-dextrose broth was made in the laboratory according to descriptions for ATCC media number 336 without agar and adjusted to pH 5.5. Nine strains of fungi used in this study were isolated from soil and preserved in our laboratory and included *Absidia coerulea* 118 (abbreviation: F1), *Aspergillus oryzae* A4 (F2), *Cunninghamella echinulata* 102 (F3), *Cunninghamella echinulata* 2 (F4), *Penicillium commune* YW01 (F5), *Penicillum griseofulvum* A5 (F6); *Phanerochete chrysosporium* A3 (F7), *Rhizopus nigricans* A1 (F8), *Trichoderma asperellum* 72 (F9). They were all maintained on PDA plate.

### Preparation of Biosorbents

Powdered fungal biomass from each of the strains was produced in flasks containing potato-dextrose broth at 30°C using a horizontal shaker at 200 rpm for aeration. The flasks were inoculated with 3.5×10^7^ mL^−1^ spores and shaken for 5 days, except with *Phanerochete chrysosporium* for 8 days. After growth, the biomass was separated from the culture broth by filtration, and washed with generous amounts of distilled water. The washed biomass was killed by autoclaving at 121°C for 20 min and dried overnight at 50°C. The dried biomass was then ground to power in a disintegrator and sieved through a No. 60 standard sieve to obtain uniform size for the biosorption studies.

### Biosorbent Characterization

The surface areas of the biosorbents were determined using the Brunauer-Emmett-Teller (BET) method at 77°K using liquid nitrogen [18]. The measurements were carried out with an Autosorp-1-C instrument, Quantachrome. The total pore volume and average pore size were determined at a relative pressure (P/P_0_) of 1. Chemical analyses of nitrogen, carbon and hydrogen contents of biosorbents were carried out using a Flash EA 112 instrument (ThermoFinnigan). Biosorbents F1, F4, F5 and F7 were selected for surface morphologies and FTIR analysis based on the biosorption capacities for Reactive Black 5 and the extreme values of BET surface area and chemical element compositions. Surface morphologies of four selected biosorbents were determined by Scanning Electron Microscopy at 20 kV and 6,000 magnifications after coating with thin layer gold under reduced pressure [19]. Fourier transform infrared spectroscopy (FTIR) spectra of virgin and dye-loaded biosorbents F1, F4, F5 and F7 were recorded using a Nicolet 5700 spectrophotometer (Thermo, USA) in the 400 to 4,000 cm wavelength region.

### Biosorption Experiments

Stock solutions (1000 mg L^−1^) of dye were prepared in deionized and double distilled water and diluted to get the desired concentration of dyes. Calibration curve for Reactive Black 5 were prepared by measuring the absorbance of different concentrations at 598 nm. Biosorption experiments were carried out with 100 ml dye solutions in 250 ml Erlenmyer flasks to which 0.1 g of biosorbent was added. The mixtures were shaken on a horizontal shaker at 200 rpm at 30°C for 6 h unless otherwise stated. The influence of hydrogen ion concentration on biosorption was first studied over the pH range from 1.0 to 9.0, with adjustments made using 0.1 M HCl or 0.1 M NaOH. Biosorption isotherms were then studied for dye concentrations ranging from 50 mg L^−1^ to 250 mg L^−1^ at the optimum pH determined for each biosorbent. The effect of contact time was investigated at different intervals over a 6 h period for each biosorbent. All the experiments were carried out in triplicate.

The biosorption capacity, *Q_e_* (mg g^−1^), was calculated as follows:
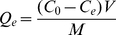
(1)where *C_o_* and *C_e_* are the initial and final concentrations (mg L^−1^), respectively, *M* is the adsorbent dosage (g) and *V* the volume of solution (L).

### Mathematical Modeling Study

Langmuir [20] and Freundlich [21] adsorption isotherms were selected to explicate the dye-fungus system in this study. The pseudo-second-order [22] and Weber-Morris [23] kinetic models were applied to provide information that are important for evaluating the efficiency of the biosorption process. The pseudo-first-order model was discarded due to its poor fit with the experimental data (R^2^<0.80 for all the tested conditions, data were not shown).

Artificial neural network models (ANN) have been used in prior research to investigate the sensitivity analysis and model the effect of parameters that affect adsorption or degradation of dyes [1], [24], [25]. The data sets were randomized and divided into training, validation and test subsets, which included 135, 45 and 45 samples, respectively. Levenberg-Marquardt (LM) which was an algorithm for least-squares estimation of nonlinear parameters and had been proved to be the fastest and the most robust was used as training method in this study [26]. The sensitivity analysis were analyzed by Garson method [27] to calculate the relative importance of the different input variables on sorption capacity. Details of the adsorption isotherms, kinetic models and ANN model analysis are provided in experimental section S1.

## Results and Discussion

### Effect of pH

Table 1 shows the biosorption capacities of Reactive Black 5 onto biosorbents as a function of pH. Maximum biosorption capacities for Reactive Black 5 onto biosorbents F1, F3 and F8 was achieved at pH 2, whereas the maximum values for the other six biosorbents were obtained at pH 1. The biosorption capacities of biosorbents decreased as the pH increased to 7. The acidic conditions could be favorable for the biosorption between Reactive Black 5 and the biosorbents due to the electrostatic attraction that exists between the positively charged surfaces of the biosorbents under acidic conditions and the negatively charged anionic dyes [28]. Under basic conditions, the presence of excess OH^−^ competed with the anionic dyes for biosorption sites, which led to low biosorption for all of the biosorbents. All the biomass materials exhibited relatively high biosorption capacities at pH 1.0–3.0. Biosorbent F1 was the most efficient for the removal of Reactive Black 5 over the tested pH range. In contrast, only 0.65 mg g^−1^ of dye could be adsorbed by biosorbent F2 at pH 9.0, which was the lowest value over the range of tested conditions.

**Table 1:** The effect of pH on the biosorption capacities of nine biosorbents for Reactive Black 5.

Biosorbent	Biosorption capacity for pH (mg g^−1^)								
	1	2	3	4	5	6	7	8	9
F1	97.75±0.07	98.24±0.08	97.91±0.06	54.10±1.16	41.27±0.32	33.94±0.35	21.20±0.28	32.24±0.26	24.78±0.29
F2	33.53±0.75	30.89±0.63	24.91±0.33	9.46±0.29	2.27±0.08	1.71±0.21	0.74±0.17	1.12±0.36	0.65±0.14
F3	96.38±0.15	97.09±0.05	72.98±0.10	31.44±0.43	22.47±0.58	20.46±0.42	15.48±0.50	19.39±0.74	18.55±0.51
F4	95.92±0.04	93.21±0.04	66.17±0.14	24.48±0.93	16.02±0.97	13.66±1.04	9.11±0.29	14.18±0.70	5.97±0.35
F5	37.57±0.29	35.34±0.07	29.17±0.41	11.96±0.62	3.34±0.40	1.20±0.16	1.16±0.23	1.34±0.29	1.30±0.21
F6	72.35±0.28	70.84±0.12	57.79±0.42	24.05±0.42	8.63±0.51	5.65±0.32	4.51±0.79	5.39±0.28	5.13±0.42
F7	65.35±0.06	64.81±0.48	50.98±0.53	11.50±0.37	4.87±0.37	2.78±0.50	2.79±0.74	3.91±0.27	3.75±0.43
F8	97.74±0.08	98.05±0.03	82.90±0.24	15.23±0.14	6.87±0.43	5.09±0.45	3.35±0.37	5.99±0.24	4.26±0.48
F9	33.59±0.39	30.85±0.61	25.38±0.21	12.51±0.27	4.64±0.29	1.85±0.12	1.12±0.21	2.93±0.28	2.50±0.22

### Biosorption Isotherms

The biosorption capacities of the powdered fungal biomass increased with increasing dye concentrations over the range from 0 to 250 mg L^−1^ (see Figure S1). The initial dye concentration provided the driving force to overcome mass transfer resistances of the dyes between the aqueous and solid phases [29]. Maximum biosorption capacity of Reactive Black 5 was 179.26 mg g^−1^ for F1 at an initial dye concentration 250 mg L^−1^, which was 5 times greater than that achieved with biosorbent F9. Biosorption isotherm models were evaluated for their ability to fit the equilibrium data. The parameters for each biosorbent obtained for the different isotherm models are listed in Table 2 along with their coefficients of determination. All the determination coefficients of Langmuir model were above 0.90, indicating that Reactive Black 5 formed a monolayer covering the biosorbents. Likewise, the determination coefficients using the Freundlich isotherm for F2, F5, F6, F7 and F9 were above 0.90, indicating both homogeneous and heterogeneous distribution of active sites on the surface of five of the biosorbents. However, sorption data generated for the biosorbents that had the highest sorption capacities (>100 mg g^−1^) failed to fit the Freundlich isotherm model (R^2^<0.80), indicating that homogeneous sorption only occurred for the sorbents with low binding capacity (F1, F3, F4 and F8). The R^2^ values for curves generated using the Langmuir model were higher than those obtained with the Freundlich isotherm model. Therefore, the Langmuir model was used for the final data analysis. All the values of Hall separation factor for the nine biosorbents were in the range from 0–1, indicating that the biosorption process was favorable. Similar patterns have been reported for adsorption of reactive dyes on to clinoptilolite [30] and for biosorption of acid dyes on to killed biomass of *Penicillium* [1].

**Table 2:** The Biosorption isotherm parameters for the biosorption of Reactive Black 5 onto biosorbents.

Biosorption isotherms		Biosorbents								
		F1	F2	F3	F4	F5	F6	F7	F8	F9
Langmuir constants	*Q_max_*(mg g^−1^)	179.26	35.21	125.95	140.84	38.41	77.62	70.52	157.82	34.18
	*K_l_* (L mg^−1^)	0.63	0.72	0.68	0.18	0.98	0.99	0.74	0.88	0.90
	R^2^	0.920	0.973	0.920	0.943	0.963	0.929	0.941	0.981	0.980
Freundlich constants	*n*	5.75	30.41	7.28	4.83	41.44	10.40	10.99	6.53	45.68
	*K_F_*(L g^−1^)	86.68	35.76	69.12	53.12	33.92	50.65	46.42	84.51	30.46
	R^2^	0.682	0.928	0.739	0.756	0.930	0.913	0.922	0.753	0.919

Reactive Black 5 biosorption capacities of different kinds of adsorbents reported in the literature are summarized in Table 3. The biosorption capacity of fungal biomass in our study was found to be comparable to and moderately higher than those of many adsorbents such as powdered activated carbon, modified sepiolite and so on.

**Table 3:** Recent reported adsorption capacities (mg/g) for Reactive Black 5.

Adsorbent	adsorption capacities (mg/g)	Literatures
Powdered activated carbon	58.82	[14]
Afsin-Elbistan fly ash	7.94	[14]
*Penicillium restrictum*	142.04	[15]
Surfactant-Modified Zeolite	12.93	[16]
Modified sepiolite	120.5	[37]
Modified zeolite	60.5	[37]
Sunflower seed shells	0.87	[38]
Mandarin peelings	0.75	[38]
seaweed *Laminaria* sp	101.5	[39]
Modified barley straw	251.92	[40]
Powdered Fungal biomass	34.18–179.26	This study

### Biosorption Kinetics

The rates of adsorption of Reactive Black 5 by the nine tested biosorbents were initially fast and then gradually decreased until equilibrium between bound and free dye was attained (See Figure S2). According to the determination coefficients (Table 4), the data were best fit using a pseudo-second-order model which gave R^2^>0.99 for all of the biosorbents. The *Q_max_* values estimated from the pseudo-second-order kinetic model were also in accord with the experimental data (*Q_eq,exp_* values in Table 4). The results suggested that boundary layer resistance was not a rate limiting step since the dye biosorption followed pseudo-second order kinetics [11]. The initial biosorption rate for biosorbent F1 was 46.7 mg g^−1^ min^−1^, which was the fastest binding rate among the tested materials. This was followed by biosorbents F7 (43.1 mg g^−1^ min^−1^) and F8 (39.4 mg g^−1^ min^−1^) (See Table S2). In contrast, the initial biosorption rates for biosorbents F6 and F9 were only 10.5 and 7.3 mg g^−1^ min^−1^, respectively. There was no apparent relationship between biosorption rate and biosorption capacity, which had a Pearson correlation coefficient of 0.675. This phenomenon may be due to differences in the chemical and textural properties of the different biosorbents. Parameters derived from the Weber-Morris model indicated that the intraparticle diffusion of Reactive Black 5 within the biosorbents occurred in two stages involving macropore diffusion followed by micropore diffusion. Since the *k_w,2_* values of nine biosorbents were smaller than the *k_w,1_* values (Table 4), the intraparticle diffusion was predicted to be the rate limiting step for biosorption of Reactive Black 5 on to the biosorbents.

**Table 4:** Parameters of pseudo-second-order and Weber-Morris model for adsorption of Reactive Black 5 onto biosorbents.

Biosorbents	*Q_eq,exp_* (mg g^−1^)	Pseudo-second-order kinetic model			Initial linear portion (Weber-Morris)			Second Linear portion (Weber-Morris)		
		*k_2_x10^−2^*(g mg ^−1^ min^−1^)	*Q_e_,_cal_* (mg g^−1^)	R^2^	*K_w,1_*	*I_1_*	R^2^	*K_w,2_*	*I_2_*	R^2^
F1	98.981	0.467	100.000	1.000	5.349	58.584	0.906	0.047	98.148	0.902
F2	33.849	1.170	34.130	0.999	1.432	22.403	0.913	0.069	32.594	0.922
F3	95.859	0.197	97.087	0.999	6.657	40.492	0.947	0.316	90.228	0.908
F4	95.908	0.265	97.087	0.999	7.807	36.748	0.905	0.126	93.636	0.935
F5	37.327	1.267	37.453	1.000	1.716	24.421	0.901	0.068	36.063	0.985
F6	72.793	0.191	74.074	0.999	4.050	35.001	0.965	0.550	63.078	0.904
F7	64.928	1.022	64.935	1.000	1.210	54.476	0.911	0.112	62.910	0.916
F8	98.754	0.394	100.000	1.000	5.266	58.376	0.904	0.152	96.051	0.915
F9	33.816	0.627	34.129	0.999	1.922	17.915	0.903	0.174	30.649	0.936

### Artificial Neural Network Modeling

The textural characteristics and chemical element composition of the biosorbents are shown in Table 5. To further investigate the degree to which different variables affected the biosorption capacities of the powdered fungal biomass, a background propagation artificial neural network (ANN) was used to conduct a sensitivity analysis on the individual variables (See Table S3). The ANN model showed satisfactory fits for the experimental data (See Figure S3). Table 6 showed that pH was the most influential parameter with a relative importance of 22.5%, followed by nitrogen content of biosorbents (15.7%), initial dye concentration (14.5%) and carbon content of biosorbents (10.1%). This indicated that biosorption is a complex process in which five factors each explained greater than 10% of the binding data. The sum total of operating parameters (pH, dye concentration, time) was 43.7%, whereas chemical element composition of biosorbents (Nitrogen content, carbon content, hydrogen content) determined 33.8% of binding capacity. Textural characteristics of the biosorbents (BET area, pore volume, pore diameter) explained 22.5%, indicating that chemical element compositions were more important than differences in textural characteristics.

**Table 5:** Textural characteristics and chemical element compositions of biosorbents.

Properties	Biosorbents								
	F1	F2	F3	F4	F5	F6	F7	F8	F9
Textural characteristics									
BET area (m^2^/g)	0.1789	0.2282	0.1928	0.06979	0.4358	0.2971	0.7656	0.2214	0.2061
Pore volume (m^3^/g)	0.0007153	0.0006945	0.0004054	0.0001616	0.002344	0.001191	0.002361	0.0005918	0.0005641
Pore diameter (nm)	7.997	6.087	4.205	4.632	10.76	8.018	6.168	5.347	5.475
Chemical element compositions									
Nitrogen content (%)	4.43	2.38	2.77	2.29	3.16	4.02	4.70	4.05	2.71
Carbon content (%)	53.97	55.24	59.03	60.21	45.79	50.51	47.73	50.32	51.48
Hydrogen content (%)	8.36	8.68	8.99	9.18	7.19	7.75	7.36	7.87	7.96

**Table 6:** The relative importance of input variables by ANN.

Input variables	Relative importance
pH	22.5%
Initial dye concentration	14.5%
Time	6.7%
BET area	10.0%
Pore volume	6.2%
Pore diameter	6.3%
Nitrogen content	15.7%
Carbon content	10.1%
Hydrogen content	8.0%

### Modeling the Effect of Textural Characteristics and Chemical Element Composition on Biosorption

**Figure 1:**
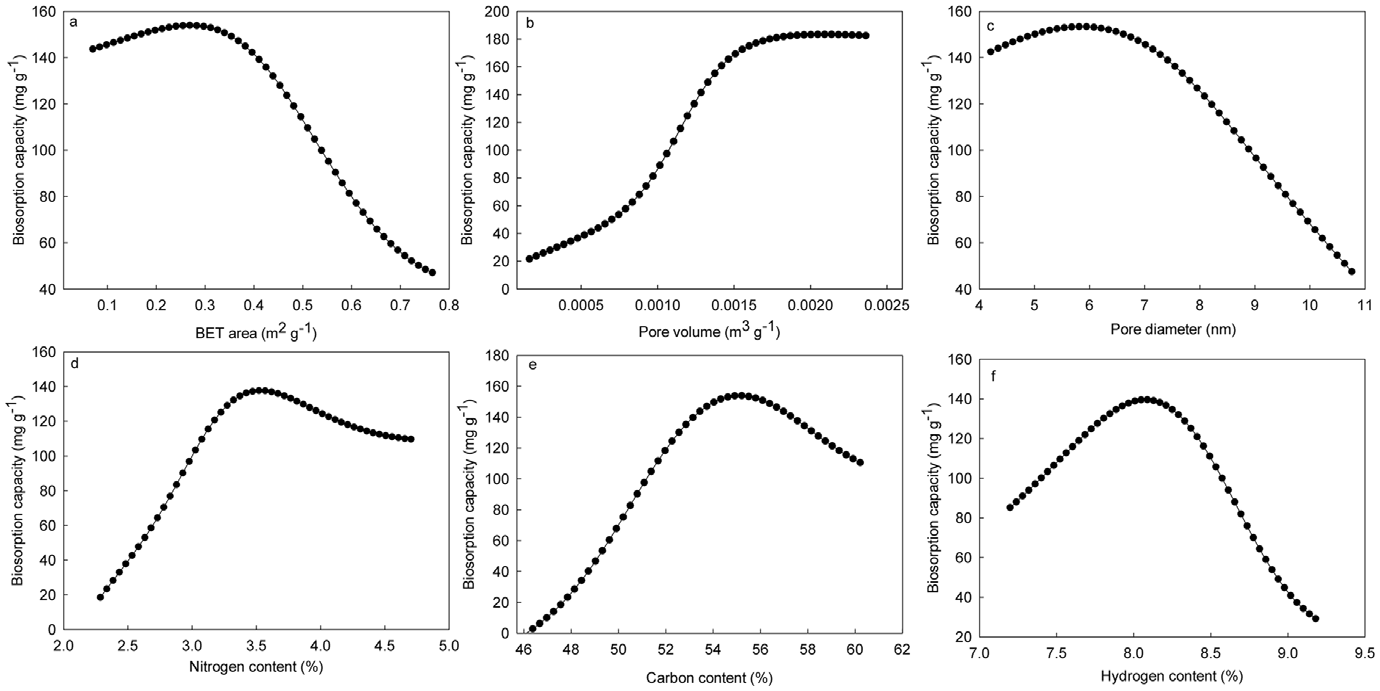
The effects of textural characteristics and chemical element compositions on biosorption as predicted using an artificial neural network model by changing the values for the variable of interest while holding the other variables constant.

**Figure 2:**
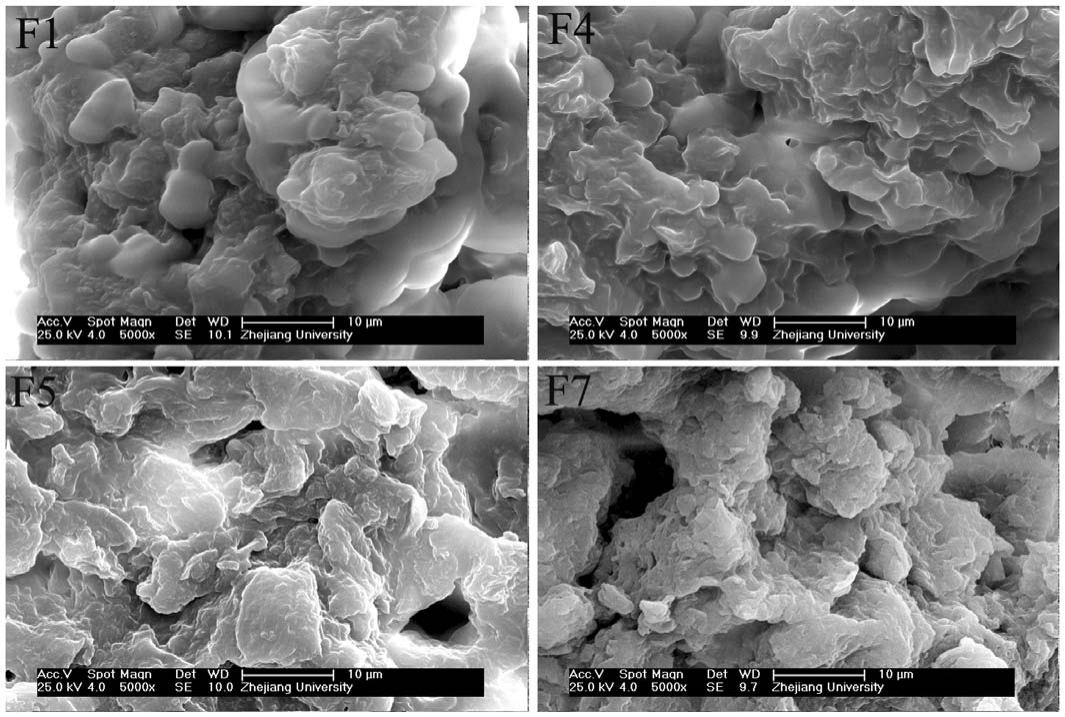
The SEM images of four selected biosorbents.

The effects of individual textural and chemical properties on biosorption capacity were examined using the ANN model. Figure 1a showed biosorption capacity first slightly increased and then decreased as the BET surface area increased from 0.238 to 0.78 m^2^ g^−1^. This was different from expectation in that adsorbents exhibiting a large surface are generally assumed to adsorb larger quantities of adsorbate than materials with lower surface areas. However, similar results have been reported in prior research [31], [32], in which surface area alone was not sufficient to predict dye adsorption capacity for biosorbents. Figure 1b showed that the biosorption capacity increased as pore volume of biosorbents increased and then reached maximum values. This infers that large pore volumes and a more porous structure favored the biosorption process. The effect of pore size on biosorption capacity is shown in Figure 1c, which shows when the average pore size ranged between 4.2–7.5 nm, the sorbents were effective for binding the dye. For larger pore sizes in the range from 7.5–11.0 nm, biosorption capacity was sharply decreased. This phenomenon may be due to the dimension of dyes aggregation in aqueous solution and the distribution of pore size of biosorbents [33]. In contrast to pore size, none of textural characteristics could be used alone to predict biosorption capacity. The SEM images of F1, F4, F5 and F7 are shown in Figure 2. Visual comparison of the images showed that the surfaces of F1 and F4 were smoother and more homogeneous than those of F5 and F7, such that the surface area and pore volumes of F1 and F4 were smaller than those of F5 and F7. Similar trend was also observed for poly (epicholorohydrin dimethylamine) modified bentonite [34] and chemical modified carbon adsorbents [35]. This is in accord with the experimental results (Table 2), which showed that the biosorption capacities for the different sorbents were not proportional to surface area.

Chemical element compositions are also important for adsorption from aqueous solutions. Figure 1d showed that the biosorption capacity increased sharply as the nitrogen content of biosorbents increased from 2.2 to 3.6%. This may be explained by the increased numbers of nitrogenous functional groups existed in the biosorbents, such as −NH_2_ and −NH– groups, which bind the dye through electrostatic interactions:
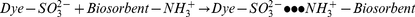
(2)Interestingly, for materials with higher nitrogen contents of biosorbents above 3.6, the biosorption capacity decreased, which may be differences in the abundance of other types of functional groups. Binding of the dye likely involves interactions between the sorbent and four 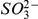 groups on the dye (Chemical structure of dye in Table S1). Similar trends were also observed for the effect of carbon content and hydrogen content of biosorbents on biosorption capacity (Figure 1e and 1f). The increase of bisorption capacity associated with increased carbon content of biosorbents may reflect differences in the abundance of carboxyl and carbonyl groups of the different biosorbents:
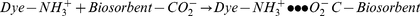
(3)Since Reactive Black 5 has four 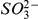 groups and only one NH_2_ group in the dye molecule, interactions between carboxyl and carbonyl groups of the biosorbents and NH_2_ group of the dyes maybe less important than that of NH_2_ groups of biosorbents and 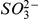 groups of Reactive Black 5. It was also observed that hydrogen content of biosorbents was inversely correlated with biosorption capacity. This may reflect decreased abundance of other functional groups in the materials with high hydrogen content. The FTIR spectra for four selected biosorbents confirmed changes in the availability of active functional groups and surface properties of the biosorbents after binding with the dye. All of the observed shifts in the FTIR spectra indicated that −NH_2_, carboxylic groups and −OH were the main functional groups that are responsible for binding Reactive Black 5 (Figure S4 and Tables S4, S5, S6, S7). Among the tested materials, biosorbent F1, which had the second highest nitrogen and fourth highest carbon content, exhibited the best binding capacity (179.26 mg g^−1^). Biosorbent F4, which had the third highest binding capacity (140.84 mg g^−1^), possessed the highest carbon content and lowest nitrogen content. Biosorbent F5, which had the lowest carbon content and the fourth lowest nitrogen content had a binding capacity of only 38.41 mg g^−1^. Biosorbent F7, which had the highest nitrogen content and the lowest carbon content exhibited 70.52 mg g^−1^ biosorption capacity for Reactive Black 5. Based on these data, carbon and nitrogen content of bisorbents as criteria for identifying superior biosorbents from fungi are better predictors of efficacy than BET specific surface area. Valix used an empirical model to find high total surface area and wider pores generally exhibited high dye adsorption capacities, and the surface chemistry of the carbon also influenced adsorption capacity significantly [36]. Compared to the results in this study, it also indicated that the selection criteria for choosing superior were significantly different from powdered activated carbon and fungal biomass.

### Conclusion

The biosorption capacities of powdered fungal biomass were in range from 34.18 to 179.26 mg g^−1^, which had competitive advantage compared to powdered activated carbon for adsorption capacity and cost. Among biosorbents in this study, the *Absidia coerulea* 118 biomass exhibited the highest biosorption capacity. The biosorption capacities were not proportional to surface areas of the sorbents, but were instead influenced by their chemical element compositions. And differences in carbon and nitrogen contents could be used as a selection index for identifying effective biosorbents. These results were significantly different from the selected criterion for selecting superior effective activated carbon. Sorption capacities of the biosorbents were not correlated with the initial biosorption rates.

### Supporting Information Available

Additional details of experimental methods, effects of initial dye concentration and time on biosorption capaicites of nine biosorbents for Reactive Black 5, the FTIR Spectra of Reactive Black 5, Unloaded and Dye-loaded Biosorbents, and information of adsorption isotherms, kinetic models and ANN model analysis are available in the Supporting Information.

## Supporting Information

Experimental Section S1 **Mathematical Modeling Study and Artificial Neural Network.**
(DOC)

Figure S1 **The effect of initial dye concentrations on biosorption capacities of biosorbents for Reactive Black 5 (30°C, 180 rpm).**
(TIF)

Figure S2 **The effect of contact time on biosorption capacities of biosorbents for Reactive Black 5 (30°C, 180 rpm).**
(TIF)

Figure S3 **The comparison between experimental values and predicted values using an ANN model.** The value of the determination coefficient (R^2^) was 0.9855, indicating the ANN could be used to fit the experimental data and investigate the effect of input variables.
(TIF)

Figure S4 **The FTIR spectrum of Reactive Black 5. 3454.4 cm^−1^: O–H stretching; 1633.6 cm^−1^: N–H bending; 1002.3–1227.7 cm^−1^: –SO_3_/C–N stretching; 739.4 cm^−1^: C–H bending.**
(TIF)

Table S1 **The Chemical structure and characteristics of Reactive Black 5.**
(DOC)

Table S2 **The initial biosorption rate of nine biosorbents for Reactive Black 5.**
(DOC)

Table S3 **Model variables and their ranges for artificial neural network.**
(DOC)

Table S4 **The FTIR Spectral Characteristics of Biosorbent F1 Before and After Biosorption of Reactive Black 5.**
(DOC)

Table S5 **The FTIR Spectral Characteristics of Biosorbent F4 Before and After Biosorption of Reactive Black 5.**
(DOC)

Table S6 **The FTIR Spectral Characteristics of Biosorbent F5 Before and After Biosorption of Reactive Black 5.**
(DOC)

Table S7 **The FTIR Spectral Characteristics of Biosorbent F7 Before and After Biosorption of Reactive Black 5.**
(DOC)
