# Supplementary material for: Computational Identification and Analysis of the Key Biosorbent Characteristics for the Biosorption Process of Reactive Black 5 onto Fungal Biomass
Source: PLoS One. 2012 Mar 19;7(3):e33551. doi: 10.1371/journal.pone.0033551 (PMC3307745; doi:10.1371/journal.pone.0033551)
Supplement: Experimental Section S1 — Mathematical Modeling Study and Artificial Neural Network. (DOC) [file pone.0033551.s001.doc]

**Mathematical Modeling Study and Artificial Neural Network**

**Mathematical Modeling Study.** Langmuir [1] and Freundlich [2] models were evaluated for their ability to fit the dye binding data.

; Langmuir model (1)

; Freundlich model (2)

where *C_e_* is the equilibrium dye concentration in the solution (mg L^-1^), *Q_e_* is the equilibrium dye uptake on the biosorbent (mg g^-1^) and *Q_max_* is the maximum biosorption capacity (mg g^-1^). *K_L_* is the Langmuir constant (L mg^-1^). *K_F_* (L g^-1^) and *n* (dimensionless) are Freundlich constants that indicate the extent of the biosorption, and the degree of nonlinearity between solution concentration and biosorption, respectively. The suitability of the biosorbents for the dyes can be expressed using the Hall separation factor (*R_L_*, dimensionless), which can be calculated by the following equation [3]: $R_{L}=\frac{1}{1+K_{L}C_{0}}$ (3) where *K_L_* and *C_0_* are the Langmuir constant (L mg^-1^) and initial dye concentration (mg L^-1^), respectively.

The equations of presudo-scond-order and Weber-Morris kinetic models were expressed as follows:

$\frac{t}{Q_{t}}=\frac{1}{K_{2}Q_{\max}^{2}}+\frac{1}{Q_{\max}}t$ Pseudo-second-order model (4)

$Q_{t}=K_{w}t^{1/2}+I$ Weber-Morris model (5) where *Q_max_* and *Q_t_* are the biosorption capacities (mg g^-1^) at equilibrium and at time *t*, respectively. *K_2_* and *K_w_* are the constants for the pseudo-second-order and Weber-Morris models, respectively. *I* is the value of intercept, which gives an idea about the thickness of the boundary layer, i.e. the larger is the intercept, the greater the boundary layer effect [4]. The initial biosorption rates of nine bisorbents were calculated using the following equation [5]:

$h=k_{2}Q_{\max}^{2}$ (6) where *k_2_* is the equilibrium rate constant of pseudo-second-order biosorption (g mg^-1^ min^-1^), Q_max_ is the maximum biosorption capacity (mg g^-1^) for the pseudo-second-order biosorption, and h is the initial biosorption rate (mg g^-1^ min^-1^).

**Artificial Neural Network.** In this study, 225 sets of data obtained from dye binding experiments on the different types of fungal biomass were used to train and test a back propagation ANN model. All data were normalized in the range 0.1-0.9 using the following equation:

(7) where min(*X_i_*) and max(*X_i_*) are the extreme values of variable *X_i_* [6]. First, we used the mean square error term (MSE) to determine the structure of the three-layered feed forward back propagation neural network ANN, which was calculated by the following equation: (8) where *y_i,pred_* and *y_i,exp_* are the values predicated by the neural network and obtained by experiments, respectively. *N* is the number of data point and *i* is an index of data. A series of topologies, in which the number of nodes was varied from 3 to 15, were used to determine the optimum number of hidden nodes. For the final model, the optimal number was 10 nodes.

The sensitivity analysis were analyzed by Garson method [7] to calculate the relative importance of the different input variables on sorption capacity, which were calculated as following:

(9) where *I_j_* is the relative importance of the jth input variable on the output variable, *N_i_* and *N_h_* are the numbers of input and hidden neurons, respectively, *W* is connection weight, the superscripts ‘*i*’, ‘*h*’ and ‘*o*’ refer to input, hidden and output layers, respectively, and subscripts ‘*k*’, ‘*m*’ and ‘*n*’ refer to input, hidden and output neuron numbers, respectively.

**Literatures cited:**

1. Langmuir I (1918) The adsorption of gases on plane surfaces of glass, mica and platinum. J Am Chem Soc 40: 1361-1403.

2. Freundlich HMF (1906) Über die adsorption in lösungen. Z Phys Chem 57: 385-470.

3. Hall KR, Eagleton LC, Acrivos A, Vermeule.T (1966) Pore- and Solid-Diffusion Kinetics in Fixed-Bed Adsorption under Constant-Pattern Conditions. Ind Eng Chem Fund 5: 212-223.

4. Özer A, Akkaya G, Turabik M (2006) Biosorption of Acid Blue 290 (AB 290) and Acid Blue 324 (AB 324) dyes on *Spirogyra rhizopus*. J Hazard Mater 135: 355-364.

5. Qin F, Wen B, Shan XQ, Xie YN, Liu T, et al. (2006) Mechanisms of competitive adsorption of Pb, Cu, and Cd on peat. Environ Pollut 144: 669-680.

6. Khataee AR, Dehghan G, Ebadi A, Zarei M, Pourhassan M (2010) Biological treatment of a dye solution by *Macroalgae Chara* sp.: Effect of operational parameters, intermediates identification and artificial neural network modeling. Bioresour Technol 101: 2252-2258.

7. Garson GD (1991) Interpreting neural-network connection weights. AI Expert 6: 46-51.
