# Supplementary figures and images for: Computational Identification and Analysis of the Key Biosorbent Characteristics for the Biosorption Process of Reactive Black 5 onto Fungal Biomass
Source: PLoS One. 2012 Mar 19;7(3):e33551. doi: 10.1371/journal.pone.0033551 (PMC3307745; doi:10.1371/journal.pone.0033551)

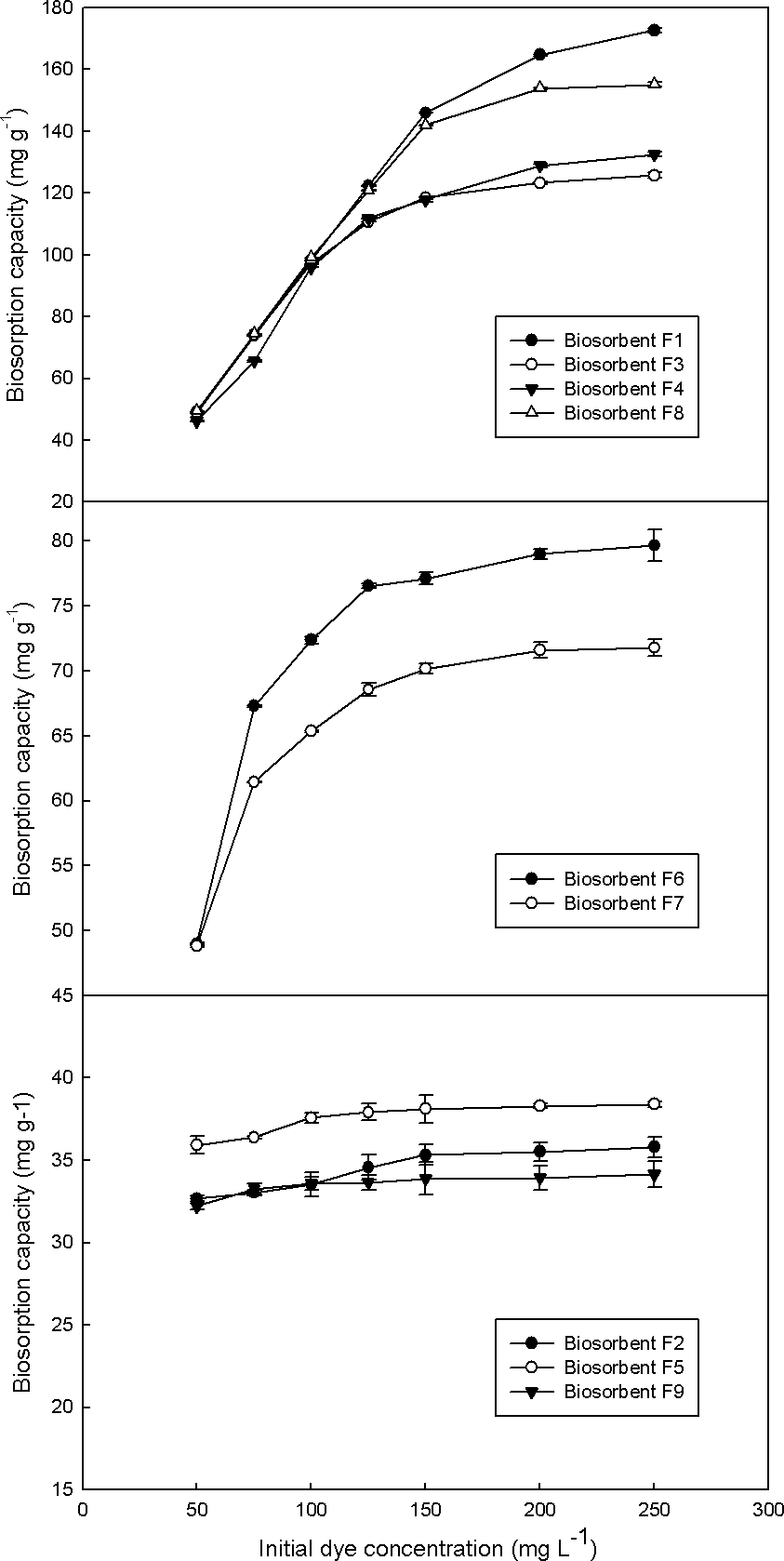

Supplement: Figure S1 — The effect of initial dye concentrations on biosorption capacities of biosorbents for Reactive Black 5 (30°C, 180 rpm). (TIF) [file pone.0033551.s002.tif]

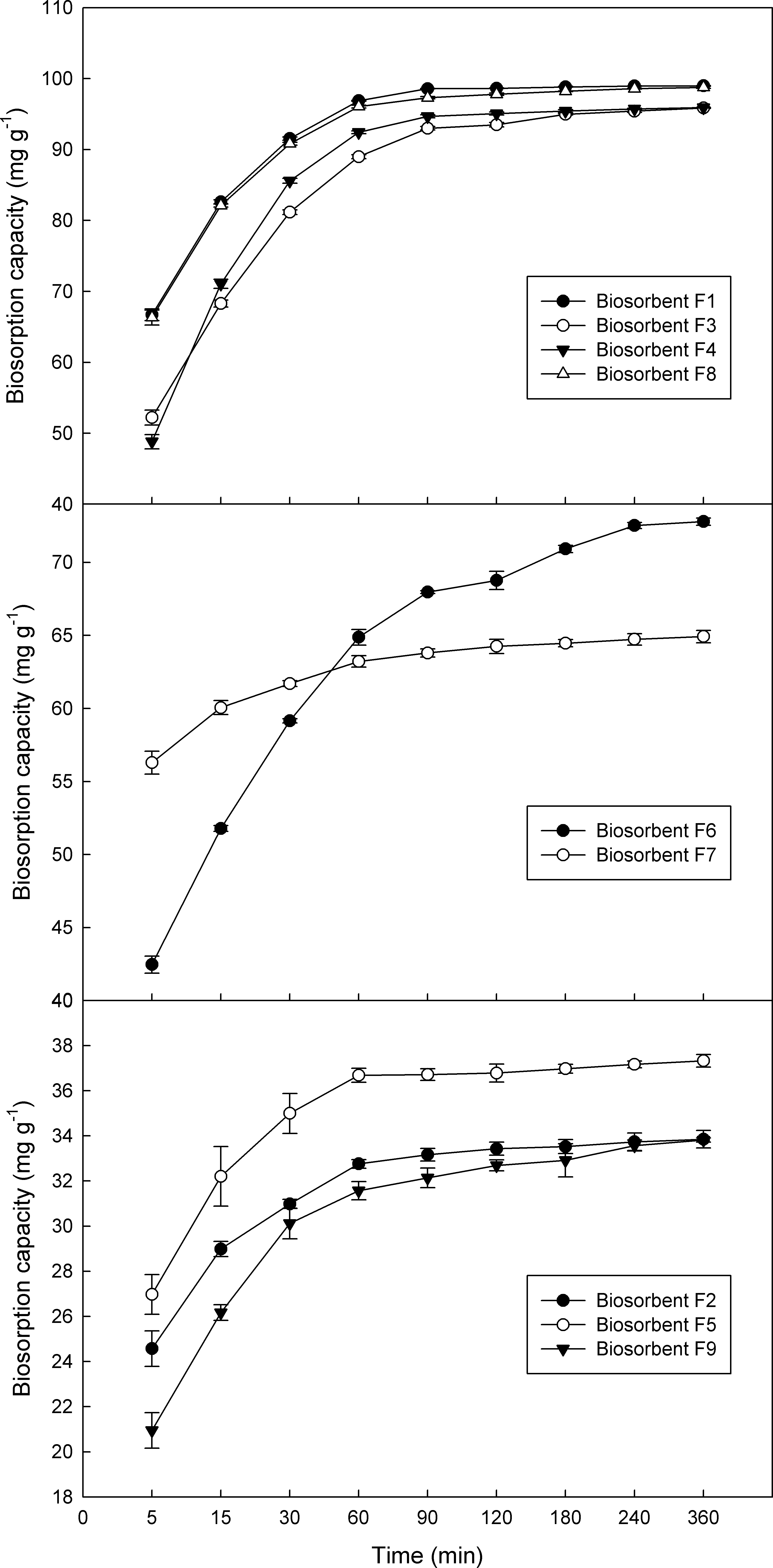

Supplement: Figure S2 — The effect of contact time on biosorption capacities of biosorbents for Reactive Black 5 (30°C, 180 rpm). (TIF) [file pone.0033551.s003.tif]

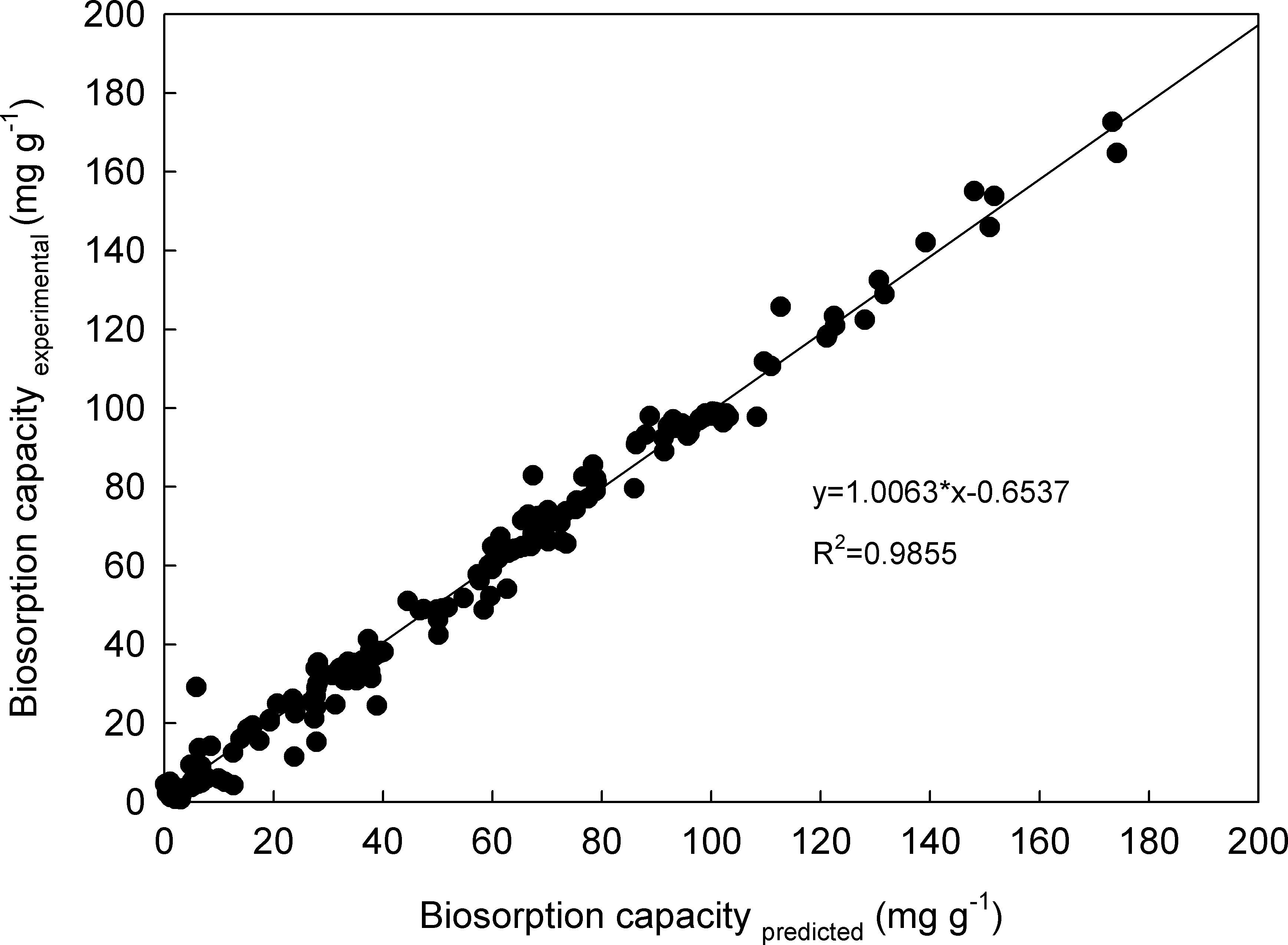

Supplement: Figure S3 — The comparison between experimental values and predicted values using an ANN model. The value of the determination coefficient (R2) was 0.9855, indicating the ANN could be used to fit the experimental data and investigate the effect of input variables. (TIF) [file pone.0033551.s004.tif]

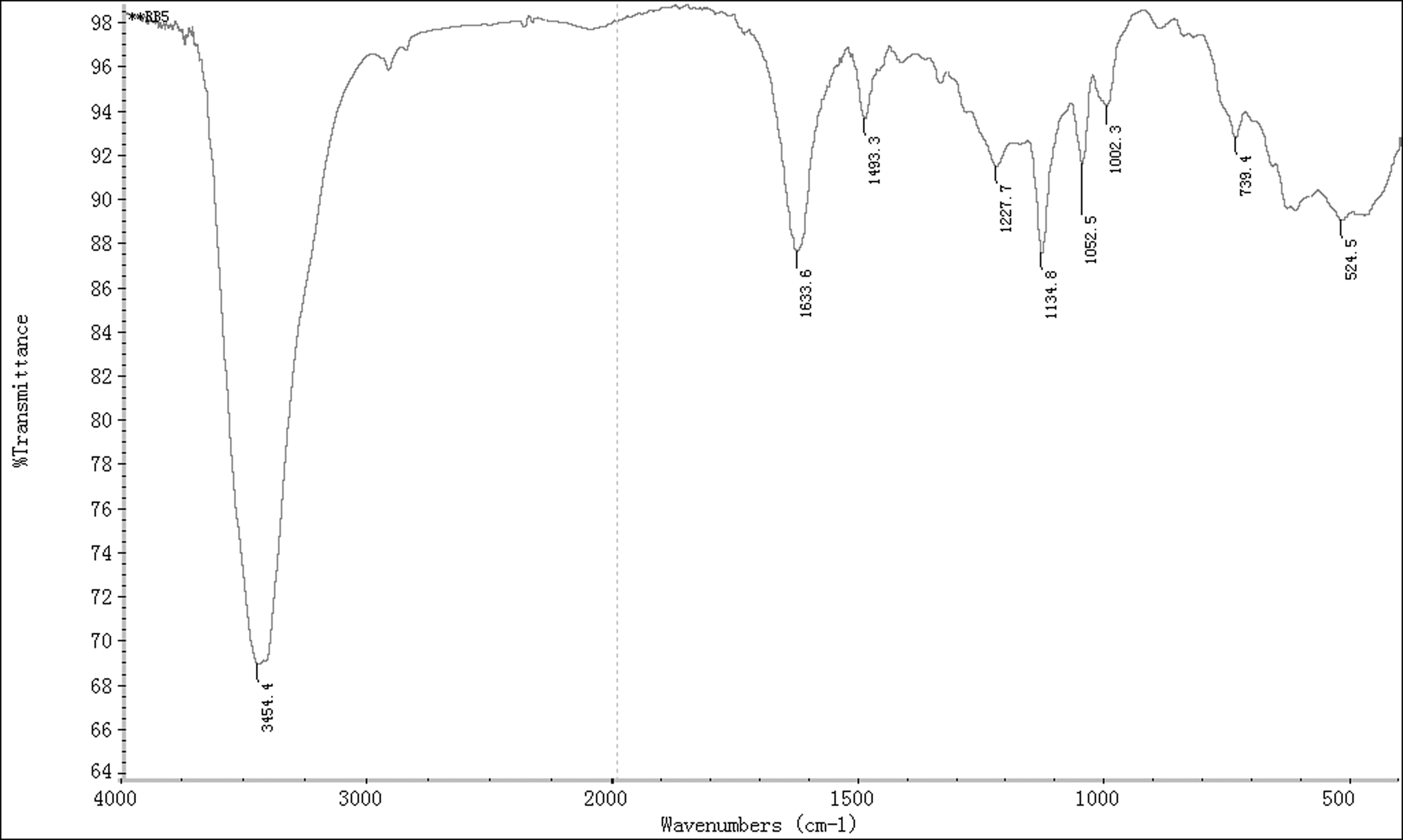

Supplement: Figure S4 — The FTIR spectrum of Reactive Black 5. 3454.4 cm−1: O–H stretching; 1633.6 cm−1: N–H bending; 1002.3–1227.7 cm−1: –SO3/C–N stretching; 739.4 cm−1: C–H bending. (TIF) [file pone.0033551.s005.tif]
