# Supplementary material for: Computational Identification and Analysis of the Key Biosorbent Characteristics for the Biosorption Process of Reactive Black 5 onto Fungal Biomass
Source: PLoS One. 2012 Mar 19;7(3):e33551. doi: 10.1371/journal.pone.0033551 (PMC3307745; doi:10.1371/journal.pone.0033551)
Supplement: Table S1 — The Chemical structure and characteristics of Reactive Black 5. (DOC) [file pone.0033551.s006.doc]

**Table S1.** The Chemical structure and characteristics of Reactive Black 5.

| Chemical structure | Color index number | λmax | Chemical class | Dye content | Molecular weight |
| --- | --- | --- | --- | --- | --- |
|  | C.I. 20505 | 598 nm | Azo dye | 55% | 991.82 |
