# Supplementary material for: Computational Identification and Analysis of the Key Biosorbent Characteristics for the Biosorption Process of Reactive Black 5 onto Fungal Biomass
Source: PLoS One. 2012 Mar 19;7(3):e33551. doi: 10.1371/journal.pone.0033551 (PMC3307745; doi:10.1371/journal.pone.0033551)
Supplement: Table S2 — The initial biosorption rate of nine biosorbents for Reactive Black 5. (DOC) [file pone.0033551.s007.doc]

**Table S2** The initial biosorption rate of nine biosorbents for Reactive Black 5.

| Biosorbents | F1 | F2 | F3 | F4 | F5 | F6 | F7 | F8 | F9 |
| --- | --- | --- | --- | --- | --- | --- | --- | --- | --- |
| Initial biosorption rate (mg g^-1^ min^-1^) | 46.70 | 13.63 | 18.57 | 24.98 | 17.77 | 10.48 | 43.09 | 39.40 | 7.30 |
