# Supplementary material for: Computational Identification and Analysis of the Key Biosorbent Characteristics for the Biosorption Process of Reactive Black 5 onto Fungal Biomass
Source: PLoS One. 2012 Mar 19;7(3):e33551. doi: 10.1371/journal.pone.0033551 (PMC3307745; doi:10.1371/journal.pone.0033551)
Supplement: Table S3 — Model variables and their ranges for artificial neural network. (DOC) [file pone.0033551.s008.doc]

**Table S3** Model variables and their ranges for artificial neural network.

| Variables | Range |
| --- | --- |
| *Input layer* |  |
| BET area (m^2^/g) | 0.0698-0.7656 |
| Pore Volume (m^3^/g) | 0.0001616-0.0024 |
| Pore diameter (nm) | 4.2050-10.76 |
| Nitrogen content (%) | 2.29-4.70 |
| Carbon content (%) | 45.78-60.21 |
| Hydrogen content (%) | 9.18-7.20 |
| pH | 1.0-9.0 |
| Dye concentration (mg L^-1^) | 50.0-250.0 |
| Time (min) | 5.0-360.0 |
| *Output layer* |  |
| Biosorption capacities (mg g^-1^) | 0.65-172.67 |
