# Supplementary material for: Computational Identification and Analysis of the Key Biosorbent Characteristics for the Biosorption Process of Reactive Black 5 onto Fungal Biomass
Source: PLoS One. 2012 Mar 19;7(3):e33551. doi: 10.1371/journal.pone.0033551 (PMC3307745; doi:10.1371/journal.pone.0033551)
Supplement: Table S4 — The FTIR Spectral Characteristics of Biosorbent F1 Before and After Biosorption of Reactive Black 5. (DOC) [file pone.0033551.s009.doc]

**Table S4 The FTIR Spectral Characteristics of Biosorbent F1 Before and After Biosorption of Reactive Black 5.**

| Wavelength range (cm^-1^) | Biosorbent F1 | | Differences | Assignment |
| --- | --- | --- | --- | --- |
|  | Before biosorption | After Biosorption |  |  |
| 3100–3500 | 3409.3 | 3439.8 | +30.5 | N–H stretching |
| 2700–2950 | 2925.2 | 2925.8 | +0.6 | –CH stretching |
| 2700–2950 | 2854.4 | 2855.1 | +0.7 | –CH stretching |
| 1750–1680 | 1745.1 | 1744.5 | -0.6 | C=O carbonyls |
| 1670–1500 | 1635.0 | 1634.1 | -0.9 | Carboxylic groups |
| 1670–1500 | 1543.7 | 1543.7 | 0 | Carboxylic groups |
| 1490–1350 | 1460.4 | 1460.1 | -0.3 | –CH bending vibrations |
| 1490–1350 | 1379.1 | - | - | –CH bending vibrations |
| 1300-1000 | 1238.3 | - | - | –SO_3_ stretching |
| 1350-1000 | 1155.7 | - | - | O–H alcohols ( primary and secondary ) and aliphatic ethers |
| 1300–1000 | 1078.6 | 1079.6 | +1.0 | C–O stretching of COOH |

From the difference of FTIR spectrum, it could be clearly delineated the major involvement of functional groups like –NH_2_,–OH, –SO_3_, –CH and –COOH present on the biosorbent F1 surface in the Reactive Black 5 biosorption process.
