# Supplementary material for: Computational Identification and Analysis of the Key Biosorbent Characteristics for the Biosorption Process of Reactive Black 5 onto Fungal Biomass
Source: PLoS One. 2012 Mar 19;7(3):e33551. doi: 10.1371/journal.pone.0033551 (PMC3307745; doi:10.1371/journal.pone.0033551)
Supplement: Table S5 — The FTIR Spectral Characteristics of Biosorbent F4 Before and After Biosorption of Reactive Black 5. (DOC) [file pone.0033551.s010.doc]

**Table S5 The FTIR Spectral Characteristics of Biosorbent F4 Before and After Biosorption of Reactive Black 5.**

| Wavelength range (cm^-1^) | Biosorbent F4 | | Differences | Assignment |
| --- | --- | --- | --- | --- |
|  | Before biosorption | After Biosorption |  |  |
| 3100–3500 | 3406.5 | 3439.9 | +33.4 | N–H stretching |
| 2700–2950 | 2925.2 | 2925.5 | +0.3 | –CH stretching |
| 2700–2950 | 2854.3 | 2854.6 | +0.3 | –CH stretching |
| 1750–1680 | 1744.2 | 1744.0 | -0.2 | C=O carbonyls |
| 1670–1500 | 1630.4 | 1632.2 | +1.8 | Carboxylic groups |
| 1490–1350 | 1461.0 | 1461.1 | +0.1 | –CH bending vibrations |
| 1490–1350 | 1376.3 | 1377.8 | +1.2 | –CH bending vibrations |
| 1350-1000 | 1158.6 | 1157.1 | -1.5 | O–H alcohols ( primary and secondary ) and aliphatic ethers |
| 1300–1000 | 1082.0 | 1083.4 | +1.4 | C–O stretching of COOH |

The changes observed in the FTIR spectrum between the unloaded and the loaded biomass indicated that functional groups including –NH_2_, carboxylic groups, and –OH groups could be responsible for the biosorption of Reactive Black 5 onto biosorbent F4.
