# Supplementary material for: Computational Identification and Analysis of the Key Biosorbent Characteristics for the Biosorption Process of Reactive Black 5 onto Fungal Biomass
Source: PLoS One. 2012 Mar 19;7(3):e33551. doi: 10.1371/journal.pone.0033551 (PMC3307745; doi:10.1371/journal.pone.0033551)
Supplement: Table S6 — The FTIR Spectral Characteristics of Biosorbent F5 Before and After Biosorption of Reactive Black 5. (DOC) [file pone.0033551.s011.doc]

**Table S6 The FTIR Spectral Characteristics of Biosorbent F5 Before and After Biosorption of Reactive Black 5.**

| Wavelength range (cm^-1^) | Biosorbent F4 | | Differences | Assignment |
| --- | --- | --- | --- | --- |
|  | Before biosorption | After Biosorption |  |  |
| 3100–3500 | 3367.7 | 3430.4 | +62.7 | N–H stretching |
| 2700–2950 | 2927.2 | 2926.9 | -0.3 | –CH stretching |
| 1670–1500 | 1635.4 | 1638.4 | +3.0 | Carboxylic groups |
| 1670–1500 | 1550.0 | 1543.9 | -6.1 | Carboxylic groups |
| 1490–1350 | 1367.5 | 1398.8 | +21.3 | –CH bending vibrations |
| 1300-1000 | 1238.1 | - | - | –SO_3_ stretching |
| 1350-1000 | 1150.9 | - | - | O–H alcohols ( primary and secondary ) and aliphatic ethers |
| 1300–1000 | 1032.2 | 1038.8 | +6.6 | C–O stretching of COOH |

The shift of functional groups bands in Table S5 indicated that –NH_2_, –CH, –SO_3_, –OH and carboxylic groups could be accounting for the biosorption of Reactive Black 5 on to biosorbent F5.
