# Supplementary material for: Computational Identification and Analysis of the Key Biosorbent Characteristics for the Biosorption Process of Reactive Black 5 onto Fungal Biomass
Source: PLoS One. 2012 Mar 19;7(3):e33551. doi: 10.1371/journal.pone.0033551 (PMC3307745; doi:10.1371/journal.pone.0033551)
Supplement: Table S7 — The FTIR Spectral Characteristics of Biosorbent F7 Before and After Biosorption of Reactive Black 5. (DOC) [file pone.0033551.s012.doc]

**Table S7 The FTIR Spectral Characteristics of Biosorbent F7 Before and After Biosorption of Reactive Black 5.**

| Wavelength range (cm^-1^) | Biosorbent F1 | | Differences | Assignment |
| --- | --- | --- | --- | --- |
|  | Before biosorption | After Biosorption |  |  |
| 3100–3500 | 3284.5 | 3404.2 | +119.7 | N–H stretching |
| 2700–2950 | 2924.2 | 2925.6 | +1.4 | –CH stretching |
| 1670–1500 | 1653.1 | 1654.1 | +1.0 | Carboxylic groups |
| 1670–1500 | 1547.4 | 1543.9 | +3.5 | Carboxylic groups |
| 1490–1350 | 1376.4 | 1403.5 | +37.1 | –CH bending vibrations |
| 1300-1000 | 1240.3 | 1231.1 | -9.2 | –SO_3_ stretching |
| 1350-1000 | 1151.9 | 1146.9 | +5.0 | O–H alcohols ( primary and secondary ) and aliphatic ethers |
| 1300–1000 | 1034.4 | 1041.2 | +6.8 | C–O stretching of COOH |

It was obviously to find that the –NH_2_, –CH, –SO_3_, –OH and carboxylic groups involved the biosorption of Reactive Black 5 onto biosorbent F7.
